# Supplementary material for: Investigating the influence of physiologically relevant hydrostatic pressure on CHO cell batch culture
Source: Sci Rep. 2021 Jan 8;11:162. doi: 10.1038/s41598-020-80576-8 (PMC7794228; doi:10.1038/s41598-020-80576-8)
Supplement: Supplementary file 1 — Supplementary Information 1. [file 41598_2020_80576_MOESM1_ESM.zip › S1/IgG/Jan17 standard curve/2/6.pdf]

===

Acq. Operator : SYSTEM   Seq. Line : 4
Sample Operator : SYSTEM
Acq. Instrument : HPLC-0039   Location : 2
Injection Date : 1/17/2019 3:14:07 PM   Inj : 1
Inj Volume : 100.000 µl
Different Inj Volume from Sample Entry! Actual Inj Volume : 60.000 µl
Sequence File : C:\Users\Public\Documents\ChemStation\1\Data\TK_CHO_IgG 2019-01-17 15-00-19\TK_CHO_IgG.S
Method : C:\Users\Public\Documents\ChemStation\1\Data\TK_CHO_IgG 2019-01-17 15-00-19\CHO_IgG_LC.M (Sequence Method)
Last changed : 12/4/2018 1:38:41 PM by SYSTEM

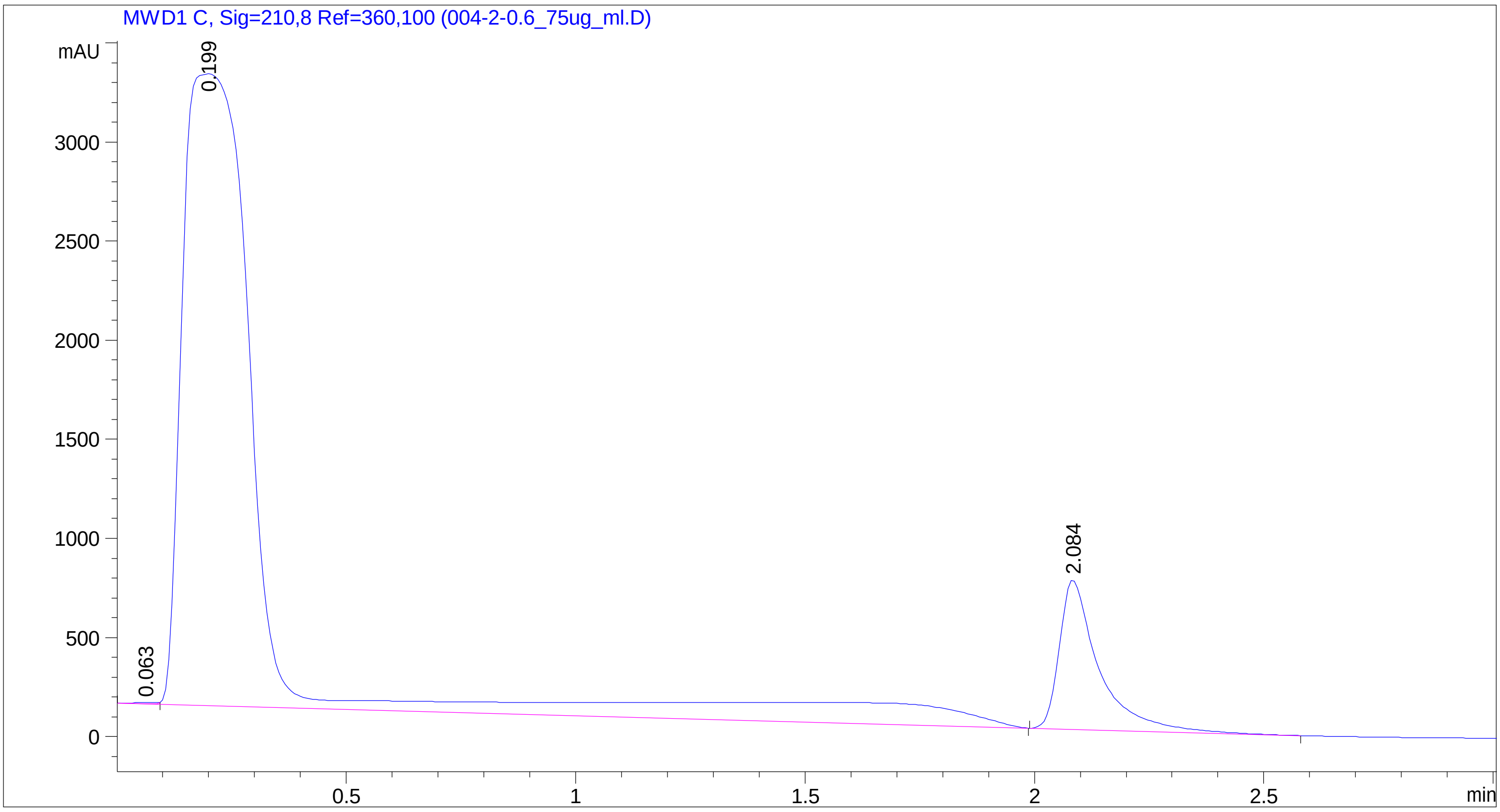

===

Area Percent Report

===

Sorted By : Signal
Multiplier : 1.0000
Dilution : 1.0000
Use Multiplier & Dilution Factor with ISTDs

Signal 1: MWD1 C, Sig=210,8 Ref=360,100

Peak #	RetTime [min]	Type	Width [min]	Area [mAU*s]	Height [mAU]	Area %
1	0.063	BV E	0.0464	25.60969	8.21202	0.0612
2	0.199	VB R	0.1826	3.73305e4	3188.60913	89.1451
3	2.084	BBA	0.0858	4519.98291	755.16455	10.7937

Totals : 4.18761e4 3951.98570

===

*** End of Report ***
